# Effects of Aerobic Training Progression on Blood Pressure in Individuals With Hypertension: A Systematic Review With Meta-Analysis and Meta-Regression

**DOI:** 10.3389/fspor.2022.719063

**Published:** 2022-02-17

**Authors:** Guilherme Tadeu de Barcelos, Isabel Heberle, Juliana Cavestré Coneglian, Bruno Allan Vieira, Rodrigo Sudatti Delevatti, Aline Mendes Gerage

**Affiliations:** Department of Physical Education, Sports Center, Federal University of Santa Catarina, Florianópolis, Brazil

**Keywords:** exercise, cardiovascular diseases, aerobic exercise, high blood pressure, health care

## Abstract

**Introduction:**

Aerobic training of moderate intensity is the primary modality recommended in the management of hypertension. The manipulation of training variables can be an important strategy for the continuity of health benefits; however, little is known about the effects of the progression of aerobic training variables in the adaptations of blood pressure in hypertensive adults.

**Objective:**

To analyze, through a systematic review with meta-analysis, the effects of aerobic training with and without progression on systolic blood pressure (SBP) and diastolic blood pressure (DBP) in hypertensive adults.

**Method:**

The search for the studies was carried out in the PubMed, Cochrane Central, SPORTDiscus and LILACS databases. Clinical trials that analyzed the effect of aerobic training, lasting at least six weeks, on blood pressure in hypertensive individuals comparing with a control group without intervention were selected. The selection of studies and data extraction were carried out independently by two pairs of researchers. The results are presented as mean difference and 95% confidence interval. Statistical significance was considered with *p* < 0.05.

**Results:**

Of the 13,028 studies found, 24 were selected and included in this review, 12 with progression of training variables and 12 without progression, with a total of 1,207 participants analyzed. There was a reduction in SBP after aerobic training with progression (−10.67 mmHg; 95% CI −15.421, −5.926; *p* < 0.001) and without progression (−10.17 mmHg; CI −12.213, −8.120; *p* < 0.001). DBP also decreased after aerobic training with progression (−5.49 mmHg; 95% CI −8.663, −2.310; *p* < 0.001) and without progression (−6.51 mmHg; 95% CI −9.147, −3.868; *p* < 0.001). According to the results of the meta-regression analyses, only age showed an association with the reduction of SBP (β: −0.323; CI −0.339, −0.307; *p* < 0.001).

**Conclusion:**

Aerobic training promotes a reduction in the SBP and DBP levels of adults with hypertension, regardless of whether or not the training variables progression.

## Introduction

Hypertension is a multifactorial chronic disease that affects more than one billion adults worldwide (Mills et al., 2016). It is considered an important cardiovascular risk factor, since it is strongly associated with the occurrence of several other cardiovascular diseases (Rapsomaniki et al., 2014) and mortality (Pan et al., 2020). In addition to medication, changes in lifestyle are essential in the treatment of hypertension and include, among other aspects, the regular practice of physical exercises (Whelton et al., 2018).

In this regard, aerobic training of moderate intensity is the primary modality recommended in the management of hypertension (class of recommendation I and level of evidence A) which should be complemented by dynamic resistance training (Brook et al., 2013; MacDonald and Pescatello, 2018; Williams et al., 2018; Barroso et al., 2020; Rabi et al., 2020; Unger et al., 2020). Previous review studies with meta-analysis showed significant mean reductions of 6.0 to 12.3 mmHg in systolic blood pressure (SBP) and 3.4 to 6.1 mmHg in diastolic blood pressure (DBP) in response to aerobic training in hypertensive individuals (Cornelissen and Smart, 2013; Igarashi et al., 2018; Cao et al., 2019). However, despite including studies with different protocols, these review studies did not analyze the influence of the progression of training variables on blood pressure (BP). Training progression can be defined as a gradual and systematic increases in training stress to maintain overload and, thus, promote continued training adaptations. As fitness improves, frequency, intensity and/or volume must be increased to induce further adaptations (Kasper, 2019). Even though progression is one of the training principles (Pfister et al., 2015), to maintain a physiological stimulus capable of causing adaptations, the guidelines regarding exercise prescription for clinical population indicate the exercise dosage (volume and intensity) to be adopted, lacking discuss whether and how training variables should progress over time.

In hypertension and exercise settings, the main guidelines recommend the practice of aerobic exercises at moderate intensity during 30 to 60 min a day or 150 min a week, in a frequency of four to seven times a week (Brook et al., 2013; Whelton et al., 2018; Sharman et al., 2019; Rabi et al., 2020; Unger et al., 2020). In general, it is recommended to gradually increase the load, especially the training intensity. However, this recommendation is superficial, without specifications indicating how and when to progress in frequency, intensity and/or volume to BP improvement. In terms of progression of intensity, for example, it is not clear if it is enough to increase the absolute load to maintain the same relative intensity or if it is necessary to progress, that is to increase the relative intensity also.

Cornelissen and Smart (2013), in a systematic review with meta-analysis, found that different training frequency and exercise session duration do not significantly affect the effect of aerobic training on BP, what the authors themselves considered counterintuitive as one would presume exercise training-induced BP reductions follow a dose–response relationship. In this sense, some studies that compared the effects of different levels of these variables on aerobic training have shown reductions in BP regardless of the duration or intensity applied (Börjesson et al., 2016; Costa et al., 2018; Gorostegi-Anduaga et al., 2018; Bahmanbeglou et al., 2019), contradicting some indications that the reductions in BP occur in greater magnitude with higher training intensities (Boutcher and Boutcher, 2017). Considering that higher intensities lead to significant benefits resulting from greater physiological adaptations (Boutcher and Boutcher, 2017), not only in BP but in other aspects related to health (Costa et al., 2018), it seems important to progress and reach higher relative intensities when planning a training program.

In addition, even though the practice of physical exercise with the recommended frequencies, durations and intensities causes beneficial effects, especially on cardiorespiratory fitness, the occurrence of a plateau in these effects interferes with the continuity of these benefits (Garber et al., 2011), possibly associated with greater levels of training. Thus, provide stimulus of volume and intensity proportional to the level of training of patients is important, even to maintain benefits already achieved.

The manipulation of training variables can be an important strategy for the continuity of health benefits. However, little is known about the effects of the progression of aerobic training variables (frequency, intensity and/or duration) in the health context, especially in the adaptations of BP in adults with hypertension. Thus, the objective of the present study was to analyze, through a systematic review with meta-analysis, the effects of aerobic training with and without progression in SBP and DBP of adults with hypertension.

## Methods

This study is characterized as a systematic review with meta-analysis and meta-regression of clinical trials, that is prospective studies comparing the effect and value of intervention(s) against a control in human beings (Friedman et al., 2010). The study followed the items of PRISMA (Moher et al., 2009) and was previously registered on the PROSPERO platform (CRD42020161767).

### Search for Articles

The PubMed, Cochrane Central, SPORTDiscus and LILACS databases were used to search for articles. The searches were carried out in December 2019 and there were no restrictions for the year of publication. The terms used for the search were “hypertension,” “exercise” and “blood pressure,” applied together. The Boolean operators “OR” and “AND” were used and the search was performed using the MeSH terms with their respective synonyms.

### Eligibility Criteria

Clinical trials published in Portuguese, Spanish and English, which included hypertensive adults (≥18 years old), of both sexes, who participated in a supervised and structured aerobic exercise intervention for at least six weeks were considered eligible. There were no restrictions on the modality, intensity, session duration, volume and weekly frequency of aerobic training. Clinical trials should compare at least one group with aerobic exercise with a control group without exercise. Studies that contained co-interventions linked to training (e.g., nutritional counseling) were only included if such intervention was applied to both groups (exercise and control). To be eligible, studies should provide data on SBP and DBP at rest before and after the intervention, or the difference between the pre- and post-intervention means with their respective dispersion values. Only studies that provided BP measurements under controlled conditions were eligible. All studies that combined aerobic exercise with another type of physical exercise, that presented only the value of ambulatory BP (24 h) or that included hypertensive individuals with other cardiovascular diseases (i.e. heart failure, coronary artery disease, peripheral artery disease) were excluded. The presence of other comorbidities (i.e. obesity or diabetes type 2) was not considered as exclusion criteria.

### Study Selection and Data Extraction

In the first selection step, the titles and abstracts of the studies were read by four independent researchers (G.T.B, B.A.V, I.H and J.C.C) divided into pairs. Subsequently, the selected articles were compared between the researchers of each pair. In the next step, the texts were read in full by the peers and the studies were included or excluded according to the eligibility criteria previously established. Disagreements between the two researchers of each pair regarding the inclusion or exclusion of the studies were resolved by the fifth researcher (A.M.G).

Data extraction was performed separately and independently by the same researchers, divided into pairs in the same way as in the previous steps. The extracted data were compared to avoid any error in the extraction process, with the disagreements resolved by the fifth researcher. For all studies, the extraction of data related to the characteristics of the sample included: sample size; sex; average age; body mass index (BMI); training status (according to the classification of each study); presence of comorbidities; use of medications; time of diagnosis of hypertension (time since the diagnostic of the disease—data provided by each study); nutritional co-intervention; and adverse events arising from the intervention. For the information related to the intervention, the following data were considered: time of intervention (duration of the entire aerobic training program, in weeks); modality; method (continuous or interval); session duration; weekly frequency; intensity; adherence to training (perceptual of sessions training completed); and withdrawals (dropout). In addition, the number of progressions for intensity, frequency and/or duration of the sessions was extracted. Studies that clearly reported progression in frequency, duration and/ or intensity of the session were classified as aerobic training with progression, and those that did not clearly report or did not progress in these variables were classified as aerobic training without progression. Progression of frequency was defined as any increase in the number of sessions of training during the week (e.g. from 2 times per week to 3 times per week), while progression of duration was considered as any increase in the session time (e.g. from 30 min/ session to 40 min/session). Progression of intensity was defined as any increase in the relative load (e.g. from 70% of HR_max_ to 80% of HR_max_) or an increase in the points of perceived subjective effort (e.g. from 11 to 15 of Borg scale). Absolute increase of training load for the same rate of physiological work (internal load) was considered intensity adjustment and not progression. Regarding the study outcomes, the information extracted was: SBP and DBP, with mean and measure of dispersion, for the exercise and control groups, at pre- and post-intervention.

### Analysis of Risk of Bias

The assessment of risk of bias was carried out independently by the same researchers, divided into pairs and the fifth reviewer was consulted to resolve the disagreements. The risk of bias was assessed according to the Cochrane Handbook (Higgins and Green, 2008), considering the following criteria: generation of random sequence; concealment of allocation; concealment of the assessment of outcomes; conducting analysis by intention to treat; and description of withdrawals and exclusions. The risk of bias was classified as: high risk—when methodological criteria, such as the proper generation of random sequences, were not reported or were not performed; low risk—when the methodological criteria were properly carried out; unclear risk—when there was no adequate description of the criteria, it was not possible to evaluate it as high or low risk.

### Data Analysis

The combined effect estimates for SBP and DBP were calculated using the difference between baseline and post-intervention values, with their respective standard deviation values and number of participants analyzed. Studies that presented other measures of dispersion had the values converted to standard deviation. The results of the analyses are presented as mean difference with a 95% confidence interval, and the calculations were performed using the random effects model. The statistical heterogeneity of the effects was assessed using the I^2^ inconsistency test, considering values above 50% as high heterogeneity (Higgins and Green, 2008).

Subgroup analyses were performed considering training progression (without progression, with progression, progression in intensity, progression in duration and progression in intensity and duration), sex, presence of comorbidities, use of medications, intervention period, training method (continuous or interval), modalities (walking/running, cycle ergometer, different modalities—e.g. walking and cycle ergometer together) and the training environment (terrestrial or aquatic). The meta-regression analysis was performed to investigate the influence of possible confounding factors on the responses of SBP and DBP, namely: mean age (years); BMI (kg/m^2^); users of antihypertensive drugs (%); SBP baseline (mmHg); weekly frequency (number of sessions per week); weekly duration (minutes); and intervention period (weeks).

To represent the results, a forest plot was generated, with the average difference and 95% confidence interval. Statistical significance was considered to be *p* < 0.05. All analyses were performed using the OpenMeta Analyst Software, version 10.10.

## Results

### Study Selection

**Figure 1:**
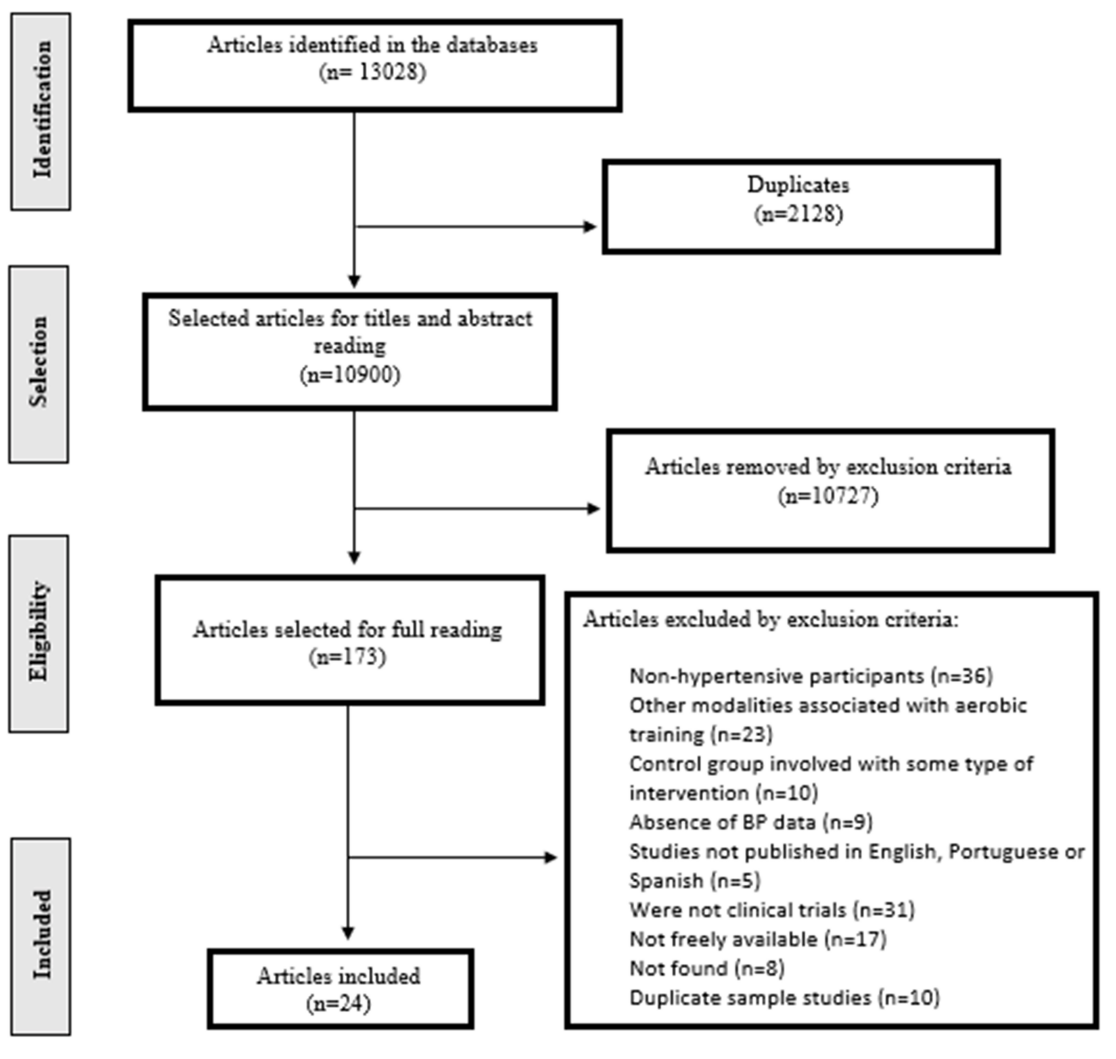
Flowchart with information on the different phases of the systematic review.

Initially, 13,028 studies were found by searching the databases. After removing duplicates, 10,900 studies were selected to read titles and abstracts. At the end of the first stage, 173 studies were selected for full reading, with 149 being excluded. Thus, 24 studies were included in the final analysis, among which 12 studies were classified as progressive aerobic training (Hagberg et al., 1989; Kokkinos et al., 1995; Tanaka et al., 1997; Turner et al., 2000; De Meirelles et al., 2009; Farahani et al., 2010; Lamina, 2010; Latosik et al., 2014; Abdelaal and Mohamad, 2015; Baghaiee et al., 2018; Wong et al., 2018; Soltani et al., 2020) and 12 studies were classified as non-progressive aerobic training (Tanabe et al., 1989; Koga et al., 1992; Miura et al., 1994; Tsai et al., 2002, 2004; Khalid et al., 2013; Arca et al., 2014; Maruf et al., 2014; He et al., 2018; Hong et al., 2018; Izadi et al., 2018; Ramos et al., 2018). In addition, four studies were analyzed twice for presenting two groups of aerobic training (Lamina, 2010; Arca et al., 2014; Soltani et al., 2020) or for collect post-intervention data at two different times (e.g. after 16 weeks of intervention and after 32 week of intervention) (Kokkinos et al., 1995) (Figure 1).

### Characteristics of the Studies

Considering all studies, 1,207 participants were analyzed, of which 716 were involved in aerobic training and 491 were part of the control group. Most of the studies included adults of both sexes (41.7%), six studies analyzed only male participants (25.0%), another six analyzed only female participants (25.0%) and two studies did not report this information (8.3%). Regarding the training status, 15 studies included untrained or sedentary participants (62.5%) and nine studies did not report this information (37.5%). The average age of the participants varied between 38.1 and 73.5 years and the BMI showed values between 23.3 and 34.4 kg/m^2^. Eleven studies included participants using antihypertensive drugs. The general information on the characteristics of the participants is shown in Table 1.

**Table 1:** Characteristics of the studies.

**References**	Sample size (% women)^**^	**Average age (years)**	**BMI (kg/m^**2**^)**	**Duration of the disease (years)**	**Trainability status**	**Comorbidities**	**Co-nutritional intervention**	**Adverse events**
**Progressive aerobic training**								
Abdelaal and Mohamad (2015)	E: 20 (60%) C: 19 (53%)	52.5	E: 34.6 ± 1.1 C: 34.1 ± 1.2	NR	Sedentary	Obesity and DM2	No	No adverse events recorded
Baghaiee et al. (2018)	E: 20 (0%) C: 20 (0%)	38.1	E: 26.8 ± 2.1 C: 27.2 ± 1.3	NR	Untrained	NR	No	NR
Farahani et al. (2010)	E: 12 (0%) C: 28 (0%)	47.7	E: 27.4 ± 4.3 C: 28.1 ± 3.5	NR	NR	No	No	NR
Hagberg et al. (1989)	E: 10 (NR) C: NR (NR)	64.4	NR	NR	NR	NR	No	NR
Kokkinos et al. (1995)	E_16_: 18 (0%) C_16_: 14 (0%) E_32_: 20 (0%) C_32_: 18 (0%)	57.5	E_16_: 31.0 ± 5.5 C_16_: 31.0 ± 5.5 E_32_: 31.0 ± 4.3 C_32_: 31.0 ± 4.3	NR	Sedentary	No	No	NR
Lamina (2010)	E_CONT_: 112 (0%) E_INT_: 140 (0%) C: 105 (0%)	58.4	E_CONT_: 25.0 ± 3.9 E_INT_: 22.5 ± 2.9 C: 24.2 ± 4.9	> 1.0	Sedentary	No	No	Unfavorable responses to training
Latosik et al. (2014)	E: 15 (100%) C: 10 (100%)	NR	E: 28.2± 6.3 C: 28.2 ± 5.5	NR	NR	No	Yes	NR
De Meirelles et al. (2009)	E: 13 (61.5%) C: 6 (66.6%)	49.5	E: 30 ± 1 C: 32 ± 2	NR	Sedentary	NR	No	NR
Soltani et al. (2020)	E_SHORT_: 10 (0%) E_LONG_: 10 (0%) C: 10 (0%)	47.9	E_SHORT_: 30.0 ± 2.3 E_LONG_: 27.3 ± 2.4 C: 29.3 ± 2.3	NR	Untrained	NR	No	NR
Tanaka et al. (1997)	E: 12 (41.7%) C: 6 (50%)	48.0	NR	NR	Untrained	Obesity	No	NR
Turner et al. (2000)	E: 11 (18.2%) C: 7 (28.6%)	66.9	E: 30.2 ± 1.8 C: 29.6 ± 1.4	E: 4.5 ± 2.7 C: 3.0 ± 1.0	Sedentary	No	No	NR
Wong et al. (2018)	E: 52 (100%) C: 48 (100%)	73.5	E: 26.0 ± 2.8 C: 26.9 ± 2.9	NR	Sedentary	NR	No	No adverse events recorded
**Non-progressive aerobic training**								
Arca et al. (2014)	E_LAND_: 19 (100%) E_WATER_: 19 (100%) C: 14 (100%)	64.0	E_LAND_: 28.3 ± 4.2 E_WATER_: 27.0 ± 5.1 C: 30.9 ± 4.8	NR	Untrained	DM2 (*n* = 5)	No	NR
He et al. (2018)	E: 20 (100%) C: 22 (100%)	57.5	E: 27.4 ± 2.1 C: 27.7 ± 2.6	NR	Untrained	No	No	NR
Hong et al. (2018)	E: 7 (0%) C: 7 (0%)	51.3	NR	NR	NR	No	No	NR
Khalid et al. (2013)	E: 12 (100%) C: 13 (100%)	52.8	E: 34.9 ± 3.5 C: 33.8 ± 4.1	NR	Sedentary	Obesity	No	NR
Izadi et al. (2018)	E: 15 (46.7%) C: 15 (40%)	61.6	E: 25.2 ± 0.6 (Men) 25.7 ± 0.7 (Women) C: 25.2 ± 0.8 (Men) 25.3 ± 1.2 (Women)	NR	Untrained	No	No	NR
Koga et al. (1992)	E: 10 (100%) C: 5 (100%)	49.0	NR	NR	NR	No	No	NR
Maruf et al. (2014)	E: 45 (NR) C: 43 (NR)	52.0	E: 27.5 ± 5 C: 25.4 ± 4.7	NR	NR	No	No	Knee joint pain (*n* = 2)
Miura et al. (1994)	E: 17 (88.2%) C: 10 (90%)	49.0	NR	NR	NR	No	No	NR
Ramos et al. (2018)	E: 12 (83.3%) C: 12 (83.3%)	60.6	E: 30.5 ± 1.5 C: 33.1 ± 2.8	NR	NR	Obesity	No	NR
Tanabe et al. (1989)	E: 21 (52.4%) C: 10 (50%)	48.9	NR	NR	NR	NR	No	NR
Tsai et al. (2002)	E: 12 (41.7%) C: 11 (54.5%)	47.9	E: 26.1 ± 4.5 C: 25.0 ± 1.8	NR	Untrained	No	No	NR
Tsai et al. (2004)	E: 52 (53.8%) C: 50 (54%)	49.1	E: 23.6 ± 1.8 C: 23.8 ± 2.2	NR	Untrained	No	No	NR

*E, exercise group; C, control group; CONT, continuous; INT, interval; NR, not reported; BMI, body mass index; DM2, diabetes mellitus 2.*

** *Tanaka et al. (1997) and Soltani et al. (2020)—Number of randomized individuals, because the number of analyzed was not reported in the study*.

### Characteristics of Interventions

#### General Characteristics

The general characteristics of the interventions is presented in Table 2. In the case of aerobic training, indoor cycling was the most reported modality (35.7%), followed by running/walking on the treadmill (25%) and soon after swimming (7.14%), aquatic exercises (7.14%) and dance (3.6%). Regarding the methods applied to training, of the 28 aerobic training groups, only five (17.9%) used interval training, while the other 23 (82.1%) used continuous training. The total duration of the interventions ranged from 6 to 37 weeks and the sessions lasted from 20 to 60 min, performed 3 to 4 times a week. Regarding the prescribed intensities, of the 28 aerobic training groups, two groups (7.14%) used percentage of VO_2_peak to prescribe exercise intensity, six groups (21.43%) used percentage of VO_2_max, 12 groups (42.86%) used percentage of HR_max_, seven groups (25%) used percentage of HR_res_ and only one group (3.57%) used both the percentage of HR_max_ and RPE in the prescription. One study reported 100% adherence of participants, four studies reported >90% adherence, two studies reported <80% adherence, and 17 studies did not report this information.

**Table 2:** Characteristics of the interventions.

**References**	**Intervention period**	**Modality**	**Method**	**Session Duration**	**Weekly frequency**	**Intensity**
**Progressive aerobic training**						
Abdelaal and Mohamad (2015)	12 weeks	Treadmill (not defined whether walking or running)	Continuous	B: 20–35 min F: 40–50 min	3	B: 60–65% HRmax F: 70–75% HRmax
Baghaiee et al. (2018)	12 weeks	NR	Continuous	B: 25 min F: 45 min	3	B: 50% HRmax F: 70% HRmax
Farahani et al. (2010)	10 weeks	Aquatic exercise	Continuous	35 min	3	B: 60–65% HRmax F: 70–75% HRmax
Hagberg et al. (1989)	37 weeks	Walking/running (Treadmill)/Cycle Ergometer	Continuous	45–60 min	3	B: NR F: 85% VO_2_max
Kokkinos et al. (1995)	16 weeks	Cycle Ergometer	Continuous	20–60 min	3	60–80% HRmax
	32 weeks					
Lamina (2010)	8 weeks	Cycle Ergometer	Continuous	B: 45 min F: 60 min	NR	B: 60% HRmax F: 79% HRmax
			Interval	B: 45 min F: 60 min	3	60–79% HRmax
Latosik et al. (2014)	8 weeks	Nordic walking	Continuous	45 min	NR	B:40–60% HRmax F: 38–69% HRmax
De Meirelles et al. (2009)	12 weeks	Walking/running	Continuous	40 min	3	B: 75% HRmax F: 85% HRmax
Soltani et al. (2020)	8 weeks	Walking/running (Treadmill)	Interval	27 min	3	B: 80% VO_2_ peak F: 100% VO_2_ peak
				32 min		B: 75% VO_2_ peak F: 90 VO_2_ peak
Tanaka et al. (1997)	10 weeks	Swimming	Continuous	B: 30 min F: 45 min	3	60% HRres
Turner et al. (2000)	28 weeks	Walking/running/Cycle Ergometer	Continuous	30-50 min	4	B: 60–70% HRmax F: 70–80% HRmax
Wong et al. (2018)	20 weeks	Swimming	Continuous	B: 25–30 min F: 40–45 min	3 to 4	B: 60% HRmax F: 70–75% HRmax
**Non-progressive aerobic training**						
Arca et al. (2014)	12 weeks	Cycle Ergometer	Continuous	20 min	3	50–60% HRres
		Aquatic exercise				
He et al. (2018)	12 weeks	Walking	Continuous	60 min	3	45–50% VO_2_max
Hong et al. (2018)	12 weeks	Walking/running (Treadmill)	Continuous	60 min	4	60% VO_2_max
Khalid et al. (2013)	8 weeks	Walking (Treadmill)	Continuous	20 min	3	60–75% HRmax
Izadi et al. (2018)	6 weeks	Cycle Ergometer	Interval	35 min	3	85–90% HRres
Koga et al. (1992)	10 weeks	Cycle Ergometer	Continuous	60 min	3	50% VO_2_max
Maruf et al. (2014)	12 weeks	Dance	Interval	35 min	3	50–70% HRres
Miura et al. (1994)	10 weeks	Cycle Ergometer	Continuous	60 min	3	40–60% VO_2_max
Ramos et al. (2018)	12 weeks	Athletics Track (not defined whether walking or running)	Continuous	50 min	3	60% HRmax/ 4–6 RPE (OMNI)
Tanabe et al. (1989)	10 weeks	Cycle Ergometer	Continuous	60 min	3	40–60% VO_2_max
Tsai et al. (2002)	12 weeks	Walking/(Treadmill)	Continuous	30 min	3	60–70% HRres
Tsai et al. (2004)	10 weeks	Walking/running (Treadmill)	Continuous	30 min	3	60–70% HRres

*B, beginning; F, final; Min, minutes; HRmax, maximum heart rate; VO_2_max, maximal oxygen consumption; VO_2_ peak, peak oxygen consumption; HRres, heart rate reserve; RPE, rating of perceived exertion*.

#### Progressive Aerobic Training

Regarding aerobic training with progression, 15 exercise groups (Hagberg et al., 1989; Kokkinos et al., 1995; Tanaka et al., 1997; Turner et al., 2000; De Meirelles et al., 2009; Farahani et al., 2010; Lamina, 2010; Latosik et al., 2014; Abdelaal and Mohamad, 2015; Baghaiee et al., 2018; Wong et al., 2018; Soltani et al., 2020) were analyzed, with the majority (80%) of the studies applying continuous training protocols. The total duration of interventions ranged from 8 to 37 weeks and the duration of sessions from 20 to 60 min, with two studies (Tanaka et al., 1997; Lamina, 2010) that progressed only in duration not showing the number of progressions made. Regarding the weekly frequency of training sessions, one study (Turner et al., 2000) reported four weekly sessions, 11 exercise groups (Hagberg et al., 1989; Kokkinos et al., 1995; Tanaka et al., 1997; De Meirelles et al., 2009; Farahani et al., 2010; Lamina, 2010; Abdelaal and Mohamad, 2015; Baghaiee et al., 2018; Soltani et al., 2020) had three weekly sessions, one exercise group (Wong et al., 2018) had a frequency of three to four weekly sessions and two (Lamina, 2010; Latosik et al., 2014) did not report this information. No study reported progression in weekly frequency. Regarding intensity, seven exercise groups (Hagberg et al., 1989; Turner et al., 2000; De Meirelles et al., 2009; Farahani et al., 2010; Latosik et al., 2014; Soltani et al., 2020) had only progression for this variable, with the most widely used method for prescribing the maximum heart rate (HR_max_), applied in 11 exercise groups (Kokkinos et al., 1995; Turner et al., 2000; De Meirelles et al., 2009; Farahani et al., 2010; Lamina, 2010; Latosik et al., 2014; Abdelaal and Mohamad, 2015; Baghaiee et al., 2018; Wong et al., 2018) ranging from 40 to 85% of HR_max_, followed by peak oxygen consumption (VO_2_peak) ranging from 80 to 100% of VO_2_peak, maximum oxygen consumption (VO_2_max) at 85%, and reserve heart rate (HR_res_), at 60%.

#### Non-progressive Aerobic Training

Regarding aerobic training without progression, 13 aerobic exercise groups (Tanabe et al., 1989; Koga et al., 1992; Miura et al., 1994; Tsai et al., 2002, 2004; Khalid et al., 2013; Arca et al., 2014; Maruf et al., 2014; He et al., 2018; Hong et al., 2018; Izadi et al., 2018; Ramos et al., 2018) were analyzed, of which only two (15.4%) used interval training (Koga et al., 1992; Maruf et al., 2014; Izadi et al., 2018). A single study showed a frequency of four training sessions per week (Hong et al., 2018), while all others used three sessions per week. The total duration of the interventions ranged from 6 to 12 weeks and the duration of the sessions ranged from 20 to 60 min. For intensity, the most used method for prescription was HR_res_, applied in six studies (Tsai et al., 2002, 2004; Arca et al., 2014; Maruf et al., 2014; Izadi et al., 2018) ranging from 50 to 90%, followed by VO_2_max, ranging from 40 to 60%, HR_max_, ranging from 60 to 75%, and rating of perceived exertion (RPE), ranging from 4 to 6.

### Analysis of the Risk of Bias

Among all the included studies, only 16.7% carried out the process of randomization and allocation confidentiality of the participants in the groups in an appropriate manner, 75% did not provide enough information to determine the randomization process and almost 80% failed to provide details regarding allocation secrecy. Still, only 25% of the studies were carried out with blinded evaluators, 58.3% of the studies described sample losses and 20.8% adopted analysis by intention to treat. Data on risk of bias separated by group with and without progression can be seen in Table 3.

**Table 3:** Risk of bias.

**References**	**Random sequence generation**	**Allocation concealment**	**Blinding of outcome assessment**	**Description of sample losses**	**Intention-to-treat analysis**
**Progressive aerobic training**					
Abdelaal and Mohamad (2015)	Low	Low	Low	Low	Low
Baghaiee et al. (2018)	Unclear	Unclear	Unclear	High	Unclear
Farahani et al. (2010)	High	Unclear	Unclear	High	Unclear
Hagberg et al. (1989)	Unclear	Unclear	Unclear	Low	Unclear
Kokkinos et al. (1995)	Unclear	Unclear	Unclear	Low	High
Lamina (2010)	Unclear	Unclear	Low	Low	High
Latosik et al. (2014)	Unclear	Unclear	Unclear	Low	High
De Meirelles et al. (2009)	Unclear	Unclear	Unclear	High	Low
Soltani et al. (2020)	Unclear	Unclear	Unclear	Low	Unclear
Tanaka et al. (1997)	Unclear	Unclear	Low	High	Unclear
Turner et al. (2000)	High	High	Unclear	Low	Unclear
Wong et al. (2018)	Low	Low	Low	Low	Low
**Non-progressive aerobic training**					
Arca et al. (2014)	Unclear	Unclear	Unclear	High	Unclear
He et al. (2018)	Unclear	Unclear	Unclear	High	High
Hong et al. (2018)	Unclear	Unclear	Unclear	High	Unclear
Khalid et al. (2013)	Low	Low	Low	Low	High
Izadi et al. (2018)	Unclear	Unclear	Unclear	Low	High
Koga et al. (1992)	Unclear	Unclear	High	High	Unclear
Maruf et al. (2014)	Low	Unclear	Unclear	Low	Low
Miura et al. (1994)	Unclear	Unclear	Unclear	Low	Unclear
Ramos et al. (2018)	Unclear	Low	Unclear	High	Unclear
Tanabe et al. (1989)	Unclear	Unclear	Unclear	High	Low
Tsai et al. (2002)	Unclear	Unclear	Unclear	Low	High
Tsai et al. (2004)	Unclear	Unclear	Low	Low	High

### Effect of Interventions

#### Effect of Aerobic Training (SBP and DBP)

The aerobic training analyzed in general, totaling 716 included participants, demonstrated a reduction in SBP with a magnitude of 10.56 mmHg (95% CI −14.083, −7.026; *p* < 0.001; I^2^: 98%) and in DBP of 5.84 mmHg (95% CI −8.226, −3.465; *p* < 0.001; I^2^: 97%).

#### Effect of Progressive Aerobic Training (SBP and DBP)

**Figure 2:**
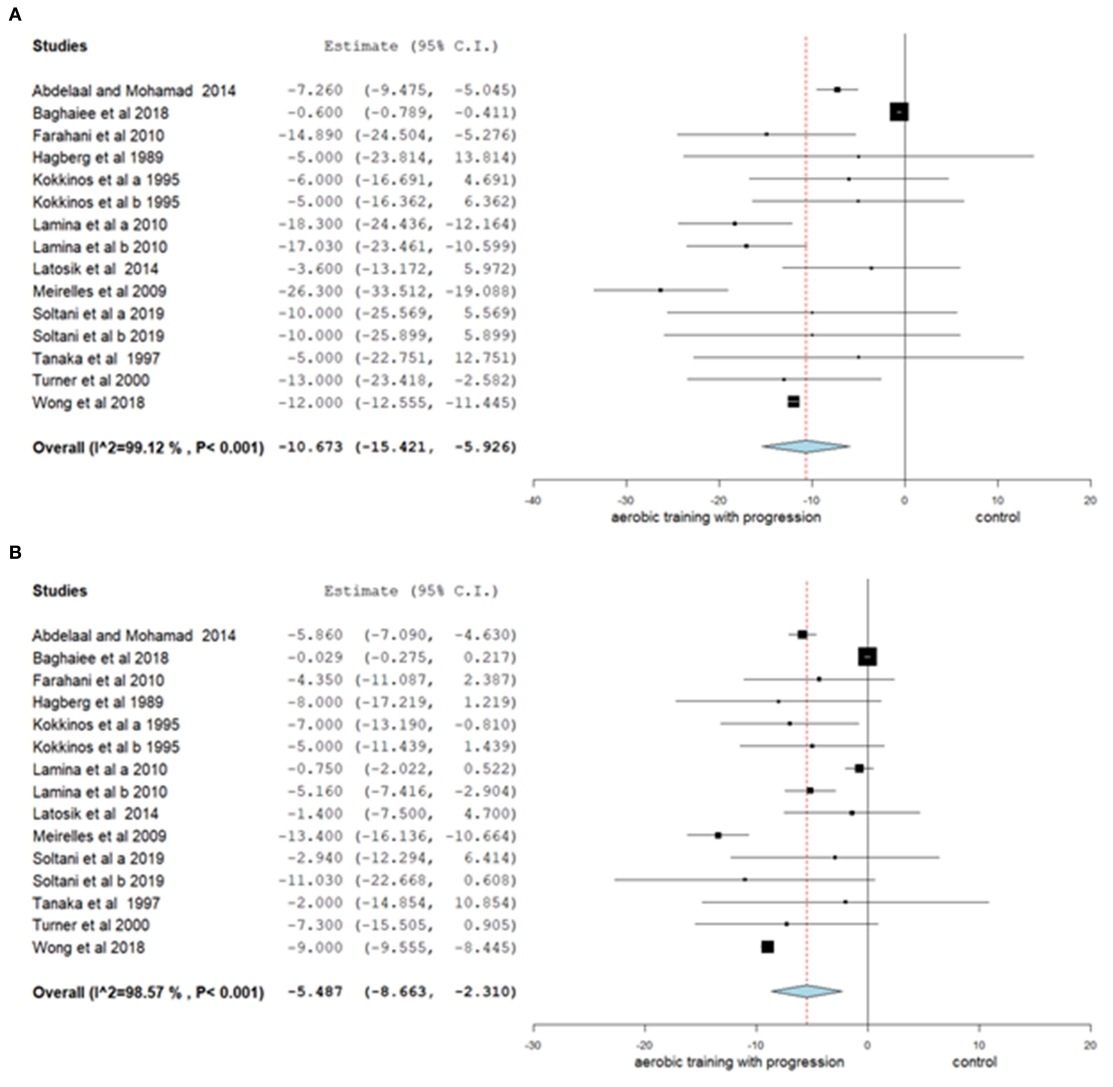
Mean differences in SBP **(A)** and DPB **(B)** observed between aerobic training with progression compared to control (without intervention). Study-specific estimates (black square); pooled estimates of random-effects meta-analyses (blue diamond). CI indicates confidence interval.

The results related to aerobic training with progression were analyzed in 15 studies, showing a reduction in SBP of 10.67 mmHg (95% CI −15.421, −5.926; *p* < 0.001; I^2^: 99%) and in DBP of 5.49 mmHg (95% CI −8.663, −2.310; *p* < 0.001; I^2^: 99%) (Figure 2).

#### Effect of Different Progressions (SBP and DBP)

Analyzing only the studies that progressed in intensity, there was a decrease in SBP of 12.89 mmHg (95% CI −20.134, −5.648; *p* < 0.001; I^2^: 64%) and in DBP of 7.09 mmHg (95% CI −11.707, −2.478; *p* = 0.003; I^2^: 69%), while for progression only in duration, a reduction in SBP of 13.98 mmHg was found (95% CI −24.238, −3.716; *p* = 0.008; I^2^: 36%) and in DBP 5.07 mmHg (95% CI −7.288, −2.843; *p* < 0.001; I^2^: 0%). When analyzing the progression in the intensity and duration variables together, statistically significant reductions were found only in the SBP (−8.28 mmHg; 95% CI −15.089, −1.479; *p* = 0.017; I^2^: 100%). In DBP, the reduction was 4.48 mmHg, without statistical significance (95% CI −9.100, 0.132; *p* = 0.057; I^2^: 99%).

#### Effect of Non-progressive Aerobic Training (SBP and DBP)

**Figure 3:**
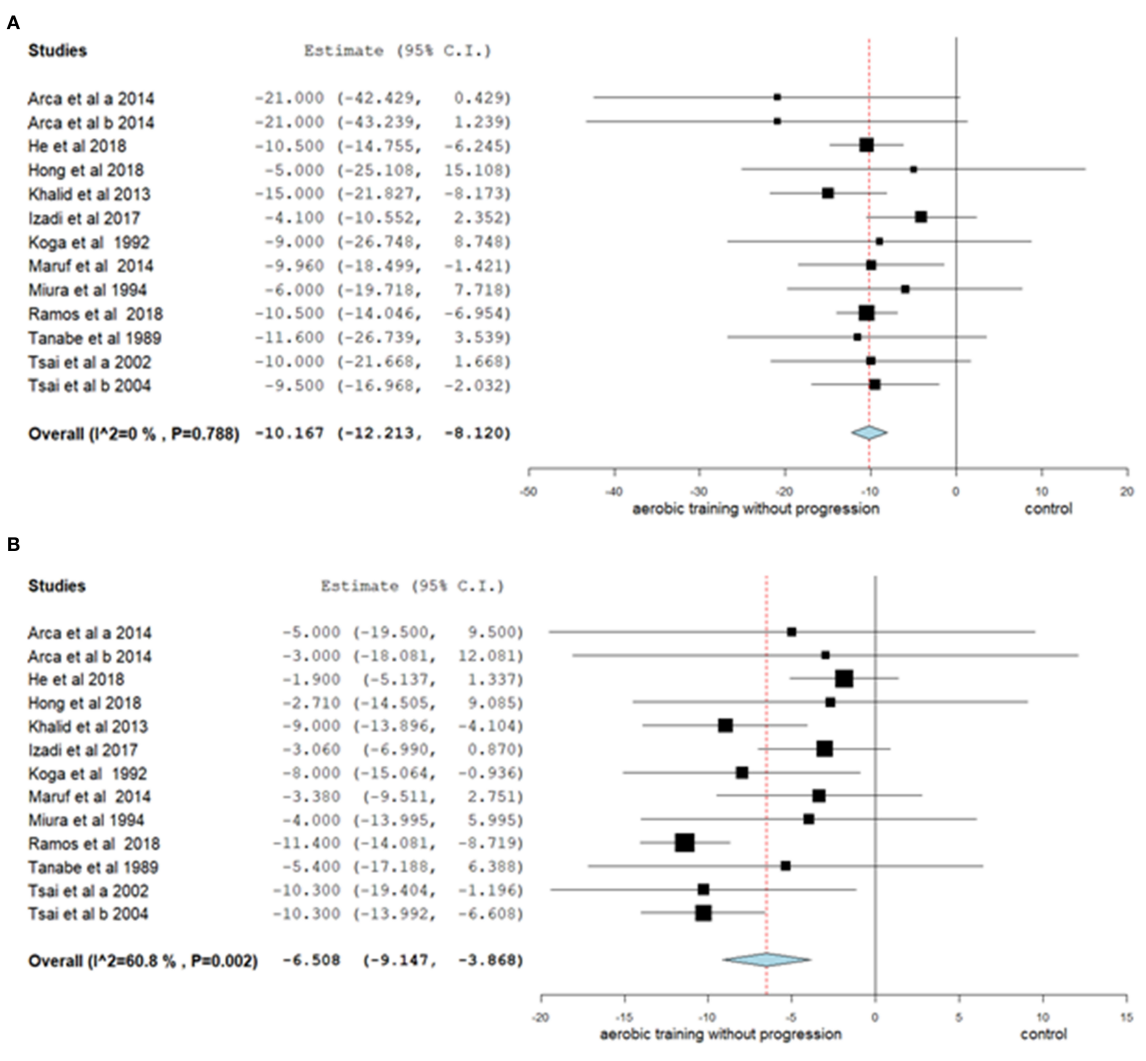
Mean differences in SBP **(A)** and DBP **(B)** observed between aerobic training without progression compared to control (without intervention). Study-specific estimates (black square); pooled estimates of random-effects meta-analyses (blue diamond). CI indicates confidence interval.

Regarding the effect of aerobic training without progression, adopted in 13 studies, a reduction was found in SBP of 10.17 mmHg (95% CI −12.213, −8.120; *p* < 0.001; I^2^: 0%) and in DBP of 6.51 mmHg (95% CI −9.147, −3.868; *p* < 0.001; I^2^: 61%) (Figure 3).

#### Effect of Aerobic Training on Subgroups (SBP and DBP)

Subgroup analyses show the effects of aerobic training on SBP and DBP separately under different conditions, between female and male, between subjects with and without comorbidities, between subjects with and without the use of antihypertensive drugs, between different weekly durations of intervention, between methods (continuous and interval), between different modalities and between aquatic and terrestrial environment.

Among the subgroups, in absolute terms, aerobic training provided the greatest reduction magnitude both in SBP (−12.01 mmHg; 95% CI −12.56, −11.46; *p* < 0.001; I^2^: 0%) and in DBP (−7.94 mmHg; 95% CI −10.58, −5.29; *p* < 0.001; I^2^: 15%) when the exercise was performed in the aquatic environment. The lowest magnitude of SBP reduction after aerobic training was observed in those individuals who did not use any antihypertensive medication (−8.18 mmHg; 95% CI −14.58, −1.78; *p* = 0.012; I^2^: 99%). For DBP, the lowest magnitude of reduction after aerobic training was observed in the subgroup of men (−2.80 mmHg; 95% CI −4.76, −0.85; *p* = 0.005; I^2^: 76%) (Table 4).

**Table 4:** Meta-analysis results.

		**Systolic blood pressure**				**Diastolic blood pressure**			
**Sub-analysis**	**N**	**Mean difference (CI 95%)**	***p* value**	**Heterogeneity**		**Mean difference (CI 95%)**	***p* value**	**Heterogeneity**	
				***I^2^***	***p* value**			***I^2^***	***p* value**
**Sex**									
Men	9	−9.89 (−17.11; −2.67)	0.007	89%	<0.001	−2.80 (−4.76; −0.85)	0.005	76%	<0.001
Women	7	−11.98 (−12.52; −11.43)	<0.001	0%	0.471	−5.81 (−9.36; −2.26)	0.001	75%	<0.001
**Comorbidities**									
With comorbidities	6	−10.17 (−13.56; −6.79)	<0.001	41%	0.132	−7.76 (−11.14; −4.37)	<0.001	67%	0.010
Without comorbidities	15	−10.46 (−13.10; −7.83)	<0.001	28%	0.153	−4.59 (−6.57; −2.61)	<0.001	64%	<0.001
**Antihypertensive drugs**									
User	10	−10.47 (−14.63; −6.31)	<0.001	63%	0.004	−6.44 (−9.95; −2.93)	<0.001	80%	<0.001
Non-user	8	−8.18 (−14.58; −1.78)	0.012	99%	<0.001	−6.45 (−11.2; −1.65)	0.008	99%	<0.001
**Intervention duration**									
<12 weeks	13	−11.37 (−14.67; −8.08)	<0.001	34%	0.110	−5.04 (−7.50; −2.57)	<0.001	71%	<0.001
12 to 24 weeks	12	−10.80 (−16.01; −5.58)	<0.001	99%	<0.001	−6.37 (−10.23; −2.52)	0.001	99%	<0.001
>24 weeks	3	−8.73 (−15.84; −1.62)	0.016	0%	0.546	−6.37 (−10.81; −1.93)	0.005	0%	0.842
**Method**									
Continuous	23	−10.62 (−14.52; −6.71)	<0.001	99%	<0.001	−6.10 (−8.78; −3.42)	<0.001	98%	<0.001
Interval	5	−10.32 (−16.08; −4.56)	<0.001	48%	0.101	−4.62 (−6.43; −2.82)	<0.001	0%	0.686
**Modality**									
Walking/running	11	−11.20 (−14.61; −7.78)	<0.001	66%	0.001	−7.65 (−10.42; −4.88)	<0.001	81%	<0.001
Cycle ergometer	9	−10.89 (−15.76; −6.02)	<0.001	51%	0.038	−4.01 (−6.31; −1.71)	<0.001	55%	0.022
Various modalities	2	−11.12 (−20.24; −2.01)	0.017	0%	0.466	−7.61 (−13.74; −1.48)	0.015	0%	0.911
**Training location**									
Dry-land	23	−11.06 (−13.53; −8.59)	<0.001	54%	0.001	−6.23 (−8.25; −4.22)	<0.001	82%	<0.001
Aquatic	4	−12.01 (−12.56; −11.46)	<0.001	0%	0.666	−7.94 (−10.58; −5.29)	<0.001	15%	0.315

#### Meta-Regression

According to the results of the meta-regression analyses, only age showed an association with the reduction of SBP (β: −0.323; CI −0.339, −0.307; *p* < 0.001), it being considered a predictor in reducing this variable as a result of aerobic training. Thus, the older the sample, the greater the reduction in SBP after the aerobic training period. The variables BMI, users of antihypertensive, baseline SBP, weekly training frequency, weekly duration and intervention period were not associated with SBP reduction. No variable was associated with a reduction in DBP due to aerobic training (Table 5).

**Table 5:** Meta-regression results.

**Moderator**	**Number of studies**	**Systolic blood pressure**			**Diastolic blood pressure**		
		***B***	**CI 95%**	***p*-value**	***B***	**CI 95%**	***p*-value**
Mean age (years)	23	−0.323	−0.339; −0.307	<0.001	−0.121	−0.337; 0.095	0.273
Body mass index (kg/m^2^)	21	0.192	−0.645; 1.028	0.653	−0.269	−0.827; 0.288	0.344
Antihypertensives users (n)	19	−0.028	−0.248; 0.193	0.805	0.071	−0.105; 0.247	0.428
SBP basal (mmHg)	28	−0.234	−0.482; 0.014	0.065	-	-	-
DBP basal (mmHg)	28	-	-	-	−0.116	−0.453; 0.220	0.498
Weekly frequency	26	−1.407	−9.057; 6.242	0.718	−1.525	−6.791; 3.741	0.570
Weekly length (min)	27	0.016	−0.045; 0.077	0.604	0.018	−0.022; 0.059	0.368
Intervention period (weeks)	28	0.107	−0.295; 0.508	0.603	−0.114	−0.366; 0.138	0.376

## Discussion

This systematic review with meta-analysis aimed to analyze the effects of aerobic training, with and without progression, in SBP and DBP of hypertensive adults. Our main results show that both aerobic training strategies (with and without progression) were effective in reducing BP and that older age is a factor associated with greater BP reductions due to aerobic training in patients with hypertension.

The general results of the present study (aerobic training vs. control group) are in line with results from other meta-analyses, which showed an average reduction of 8 to 12 mmHg in SBP and 5 to 6 mmHg in DBP in hypertensive adults (Cornelissen and Smart, 2013; Igarashi et al., 2018; Cao et al., 2019). Thus, our results reinforce the importance of aerobic training as a primary strategy for the treatment of hypertension, since the reduction in BP resulting from this practice is like that achieved with treatment with antihypertensive drugs (Naci et al., 2019). These results are clinically relevant since a 10 mmHg decrease in SBP is associated with 20% reduction in the risk of developing cardiovascular disease, 27% in the occurrence of stroke and 13% in the risk of mortality (Ettehad et al., 2016).

Although there is no difference in BP reduction between aerobic training with and without progression, reductions of greater magnitudes occurred in studies that progressed in the duration and intensity variables separately. The studies that progressed in intensity (Hagberg et al., 1989; Turner et al., 2000; De Meirelles et al., 2009; Farahani et al., 2010; Latosik et al., 2014; Soltani et al., 2020) showed reductions of 12.89 mmHg (SBP) and 7.09 mmHg (DBP) and achieved the highest percentages of intensity compared to the other studies. Studies that progressed in duration (Tanaka et al., 1997; Lamina, 2010), on the other hand, showed reductions of 13.98 mmHg in SBP and 5.07 mmHg in DBP, and achieved the longest durations in comparison to all studies with and without progression. The studies that progressed in both (Kokkinos et al., 1995; Lamina, 2010; Abdelaal and Mohamad, 2015; Baghaiee et al., 2018; Wong et al., 2018) showed more modest reductions in SBP (8.28 mmHg), with no significant reduction in DBP, and achieved session durations longer than the other studies, however the percentage of intensity was below that observed in general, both in with progression and without progression studies. Although the greatest reductions in SBP occur with studies that have progressed in duration, it is possible that the intensity of training is an important modulator of BP reduction, since both studies that have progressed in duration and those that have progressed in intensity have achieved or maintained a high intensity of training. In addition, our results showed a response of greater magnitude in studies with progression in duration, for SBP, and in intensity, for DBP, which may be associated with different mechanisms of action. In another study conducted in our group, with the objective of evaluating the effects of the progression of aerobic training on the BP of adults with diabetes, reductions in BP were also observed regardless of whether the training progressed (Heberle et al., 2021). Bearing in mind that the performance of these mechanisms in response to training is still unknown (Hellsten and Nyberg, 2015), it is not possible to state the reason associated with these differences.

The literature points to exercise intensity as a determining factor for BP changes in response to training programs (Pescatello et al., 2015; MacDonald and Pescatello, 2018), indicating a dose–response relationship, so that higher intensities of training promote greater reductions in BP (Cornelissen and Smart, 2013). As for the duration of the session, there seems to be no dose–response relationship, as longer durations do not necessarily indicate greater reductions in BP, with beneficial effects occurring even with shorter periods of exercise (Ishikawa-Takata et al., 2003). However, although some studies have investigated the effects of different intensities and durations of exercise, there is insufficient evidence to determine the relationship of these variables with the BP response (Pescatello et al., 2019), especially when not analyzed as isolated doses, but in relation to their manipulations throughout interventions. In the general analysis of our results, the greatest reduction occurred in the study by De Meirelles et al. (2009), both for SBP (−26.3 mmHg) and for DBP (−13.4 mmHg), having reported progression in intensity and reached high intensity (85% HR_max_). In addition, although there was no progression in duration, the length of the sessions was intermediate when compared to the other studies (40 min).

Although the evidence regarding progression is not concrete, the manipulation of training variables, such as duration and intensity, are recommended, and must respect a gradual process (Garber et al., 2011), especially in intensity progression (Pescatello et al., 2015). This strategy, in addition to reducing the risks of musculoskeletal injuries and cardiovascular events, favors greater participation by the participant in training (Garber et al., 2011). In addition, other precautions must be considered in this process, such as the levels of BP the person has, recent changes in antihypertensive drugs, effects caused by exercise and medications, in addition to the presence of other diseases and related conditions (Pescatello et al., 2015).

With regard to the subgroup analysis by sex, the effects of the interventions were positive for both men and women. Although the magnitude of reduction observed for women was greater than men, in absolute terms, there was no statistical difference between the groups. Turnbull et al. (2008), in a meta-analysis, also demonstrated that men and women have BP reductions of similar magnitudes. This finding suggests that the effects of aerobic training, with and without progression, on BP are similar between sexes, but it needs to be confirmed in future studies, since the majority of studies included in this review was carried out with both men and women. For analysis between practitioners with and without comorbidities, both groups showed significant BP reductions, with similar magnitudes. This finding demonstrates great clinical relevance, considering that hypertensive patients with comorbidities, which present more serious health risks and greater chances of developing coronary artery disease (CAD) (Wang et al., 2017), benefit from the training as much as those without comorbidities.

Regarding the results by users and non-users of antihypertensive drugs (no statistical difference between groups), both showed significant and similar reductions in SBP and DBP, emphasizing that aerobic training also has great hypotensive potential, being able to further optimize the treatment of hypertension, reducing the risks of complications and improving the clinical picture of hypertensive patients (Moraes-Silva et al., 2017). Reinforcing these results, the meta-regression analysis showed that the use of antihypertensive drugs was not a moderator in reducing BP during aerobic training. However, different dosages and classes of antihypertensive drugs were used in the studies included in this review, which makes it difficult to understand the effects of these factors on the pressure responses observed. Similar to our results, meta-analysis by Sardeli et al. (2020) also found that medication use did not affect BP reduction in response to training. However, this result was not specific to aerobic training and included few studies. Studies evaluating the effects of drug therapy and exercise on BP variables mainly focus on outcomes separately and have conflicting results. Thus, the evidence regarding the interaction between the use of medications and pressure responses to exercise is still scarce (Pescatello et al., 2019).

With regard to the duration of the interventions, it was shown that regardless of the aerobic training being performed for short or long periods (<12 weeks, 12–24 weeks and >24 weeks), the BP reductions are similar (no statistical difference between groups). Likewise, a systematic review by Cao et al. (2019) demonstrated that interventions of <8 weeks, between 8 and 12 weeks and of more than 12 weeks were similarly effective in decreasing BP. On the other hand, Cornelissen and Smart (2013) observed that aerobic training periods of <12 weeks produced greater reductions in SBP and DBP when compared to longer periods, which could be related to the greater adherence of participants to shorter. However, we should consider the importance of the continuity of the training, in order to maintain the benefits achieved. Considering the divergence of the available findings, there is a need for more studies with good methodological quality to better understand the role of the duration of the intervention in the non-drug treatment of hypertensive patients.

As for the training methods, the continuous and the interval methods promoted similar reductions (no statistical difference between groups) in SBP (−10.62; −10.32 mmHg, respectively) and DBP (−6.10; −4.62 mmHg, respectively), showing that both are effective. Another recent meta-analysis, carried out with the hypertensive population (Leal et al., 2020), showed reductions in SBP for both training methods when compared to control groups (continuous: −3.70 mmHg; interval: −5.64 mmHg), but without difference between training groups. For DBP, reduction was also found after continuous (−2.41 mmHg) and interval (−4.80 mmHg) training when compared to control groups, but the magnitude of DBP reduction in the interval method was significantly greater when compared to the continuous one.

Regarding the training modality, our study shows that SBP and DBP reduce in a similar way regardless of whether the aerobic exercise is performed with walking/running (−11.20 mmHg), on cycle ergometers (−10.89 mmHg) or combining the training modalities (−11.12 mmHg) (no statistical difference between groups). This finding has an important practical application, as it demonstrates that exercise professionals can choose the form of aerobic exercise according to the patient's preference, thus being able to favor adherence due to the ease of access to a certain modality or to specific clinical conditions (i.e., using a cycle ergometer due to difficulty mobility with support of their own weight), without prejudice to the reduction in BP.

As for the training location, conducting training in water may be an alternative for the hypertensive population, as it has also shown slightly higher magnitudes of BP reductions (SBP—terrestrial: −11.06 mmHg; aquatic: −12.01 mmHg; DBP—terrestrial: −6.23 mmHg; aquatic: −7.94 mmHg) in absolute terms, without statistical differences. Another study of systematic review also observed that training in water reduces SBP and DBP in a similar way to terrestrial training in adults and the elderly, with 54.5% of the sample classified as hypertensive and 27.3% with pre-hypertensive (Reichert et al., 2018). It is noteworthy that studies comparing exercises performed in different media and evaluating different outcomes in hypertensive patients are still scarce, especially in the case of chronic effects.

The meta-regression analysis showed a significant association between age and SBP responses, indicating that older individuals showed greater magnitude of reductions in SBP after aerobic training. Since the prevalence of hypertension is higher in older adults (McConnell, 2018), the results of the present study suggest that the practice of aerobic training is an important non-drug strategy for reducing BP in the elderly, which has been observed previously (Kelley and Kelley, 2018). However, our results were different from those evidenced by previous studies (Cornelissen and Smart, 2013; Thomopoulos et al., 2018; Sardeli et al., 2020). There is still disagreement in the literature regarding the influence of age on the effects of BP reduction in response to aerobic training, so that other factors must also be considered, like time of hypertension diagnostic, presence of comorbidities, complications due to hypertension and presence of target organ injury.

An important point to be highlighted in the present study is that most studies with progression used HR_max_ to prescribe the intensity of exercise (Kokkinos et al., 1995; Turner et al., 2000; De Meirelles et al., 2009; Farahani et al., 2010; Lamina, 2010; Latosik et al., 2014; Abdelaal and Mohamad, 2015; Baghaiee et al., 2018; Wong et al., 2018) while only one study without progression used this method (Khalid et al., 2013). HR_max_ is a limited method to prescribe and control intensity, because it does not consider the HR_rest_, which is influenced by factors such as age and mainly the training status of the subject. Considering this, the methods for the determination of the HR training target zone that includes HR_rest_ in the calculation, beside HR_max_, like the prescription model based on HR reserve, has greater precision. Therefore, we can explain, in part, why the studies with progression did not find greater chronic reductions in BP, since the prescribed intensity may have been underestimated in these studies. That is, not progressing violates a training principle, but using more suitable methods for prescribing intensity (such as HR reserve) seems to mitigate the effects of the lack of progression in training.

Finally, our study exposes some limitations that need to be highlighted. Although the general analysis has a considerable number of studies, some sub-analyses were carried out with a small number of studies. When assessing methodological quality, the set of studies analyzed did not clearly report most of the information, and of the five evaluation criteria, three were reported unclearly in more than 50% of the studies, making it difficult to assess the risk of bias. Another limitation is related to the lack of important information in the studies, which prevent association with the results, such as disease duration, antihypertensive drugs used and the training status of the participants. Still, the search having been carried out more than a year (~18 months) is also a limitation.

On the other hand, the present study has some strengths. As far as we know, this is a first meta-analysis that assesses the effects of the progression of aerobic training in patients with hypertension, with a significant number of participants being analyzed. The analysis of possible moderators of the effect using the meta-regression analysis is also a strong point of the study. In practical terms, although some guidelines recommend the progression of training, more precise information is lacking in relation to the way to progress. In this regard, the present study presents results that will possibly assist in the prescription of exercises, such as manipulation and the increase of the variables of the training, especially session length and intensity, thus maximizing the beneficial effects of exercise on BP.

## Conclusion

Aerobic training promotes a reduction in SBP and DBP levels in adults with hypertension, regardless of whether there is progression of the training variables. However, when manipulating the training variables, a response of greater magnitude seems to occur with the progression in duration, for SBP, and in intensity, for DBP. Nevertheless, although there is no chronic difference, the progression of the training variables must be considered, in order to potentiate the effects caused by aerobic training on the pressure response.

## Data Availability Statement

The original contributions presented in the study are included in the article/Supplementary Material, further inquiries can be directed to the corresponding author.

## Author Contributions

GTB and IH participated in the literary search, data extraction, in data analysis, interpretation of data for the work and writing of the manuscript. JCC and BAV participated in the literary search, data extraction, interpretation of data for the work and writing of the manuscript. RSD and AMG participated in the initial study design, interpretation of data for the work and critical review of the manuscript. All authors contributed to the article and approved the submitted version.

## Conflict of Interest

The authors declare that the research was conducted in the absence of any commercial or financial relationships that could be construed as a potential conflict of interest.

## Publisher's Note

All claims expressed in this article are solely those of the authors and do not necessarily represent those of their affiliated organizations, or those of the publisher, the editors and the reviewers. Any product that may be evaluated in this article, or claim that may be made by its manufacturer, is not guaranteed or endorsed by the publisher.
