# Supplementary material for: Effects of Aerobic Training Progression on Blood Pressure in Individuals With Hypertension: A Systematic Review With Meta-Analysis and Meta-Regression
Source: Front Sports Act Living. 2022 Feb 17;4:719063. doi: 10.3389/fspor.2022.719063 (PMC8891157; doi:10.3389/fspor.2022.719063)
Supplement: Supplementary file 1 [file Data_Sheet_1.DOCX]

Supplementary Material

**SEARCH STRATEGY**

((((hypertension OR “blood pressure high” OR “blood pressures high” OR “high blood pressure” OR “high blood pressures”) AND (exercise OR exercises OR “physical exercise” OR “physical exercises” OR “exercise aerobic” OR “aerobic exercise” OR “aerobic exercises” OR “exercises aerobic” OR “exercise training” OR “exercise trainings” OR “training exercise” OR “trainings exercise”) AND (“blood pressure” OR “pressure blood” OR “diastolic pressure” OR “pressure diastolic” OR “systolic pressure” OR “pressure systolic” OR “pressure systolic”))))
